# Cooking frequency and hypertension with gender as a modifier

**DOI:** 10.1186/s12937-019-0509-4

**Published:** 2019-11-29

**Authors:** Yu Zhang, Tianyu Tang, Kun Tang

**Affiliations:** 1 School of Health Humanities, Peking University Health Science Center, No. 38 Xueyuan Rd., Haidian District, Beijing, 100191 China; 2 Research Center for Public Health, Tsinghua University, Haidian District, Beijing, 100084 China

**Keywords:** Cooking, Hypertension, Gender difference, China

## Abstract

**Background:**

The effect of cooking frequency on hypertension is understudied. This study aimed to examine the effect of cooking on hypertension with a particular focus on gender differences.

**Methods:**

The present study utilized cross-sectional data from China Kadoorie Biobank with a 512,891-population of China. Hypertension was identified by established diagnosis or by the 1999 WHO/ISH Guidelines for the Management of Hypertension on examination. Cooking frequency was obtained from a self-reported questionnaire and categorized as daily cooking, weekly or monthly cooking and never cooking. Multivariable logistic regression models were employed to examine the associations between cooking frequency and hypertension in men and women, respectively. Stratified analyses by demographic and socio-economic characteristics were conducted.

**Results:**

Men who ever cooked had higher odds of hypertension compared with those who never cooked (weekly or monthly cooking adjusted odds ratio (AOR): 1.05, 95% CI: 1.02–1.07; Daily cooking AOR: 1.09, 95% CI: 1.06–1.11), while protective effects of cooking against hypertension were observed in women (weekly or monthly cooking AOR: 0.94, 95% CI: 0.89–0.99; daily cooking AOR: 0.96, 95% CI: 0.92–0.99). Socio-economic status including occupation, household income, education and region could further modify the effect of daily cooking on hypertension among men and women, respectively.

**Conclusion:**

The present study highlighted the effect of cooking on hypertension. We found the opposite trends in men and women with regards to the association between cooking and hypertension. Factors relating to socio-economic status such as education, household income and occupation could further modify the gender-specific effects. Interventions to reduce hypertension should consider the gender differences in food choice and psycho-social stress related to cooking.

## Introduction

Hypertension is one of the leading risk factors for cardiovascular diseases [1]. In China, the number of adults with hypertension increased from 594 million in 1975 to 1.13 billion in 2015 [2]. Many factors contribute to hypertension, including diet, physical activities, social stress etc. [3, 4]. Healthy lifestyles (e.g. exercising often, healthy food) have protective effects against hypertension. However, psychosocial stress, including occupational stress, economic/financial stress, stress associated with racial discrimination, depression, and anxiety, could increase hypertension risks [3, 4].

Eating home-cooked meals is believed to generate good health outcomes [5]. Frequent intake of away-from-home meals, including restaurant food and fast food, leads to weight gain [6], obesity [7, 8], and cardiometabolic risks [9, 10]. However, the healthfulness of home-prepared foods varies widely across households [11]. On the other hand, cooking at home might also negatively influence people’s health by emitting harmful fumes. Household air pollution including cooking oil fumes could result in several adverse health outcomes including lung cancer [12], elevated blood pressure [13] and coronary heart disease [14]. Household ventilation helps to alleviate these adverse outcomes [15].

Food preparation tasks are traditionally performed by women, and women also generally spend more time than men on cooking [16–18]. In traditional social contexts, housework or “domestic labor” is generally viewed as the responsibility of women rather than that of men [19]. In China, similar cultural norms were observed with women doing the majority of domestic work [20]. Shifting domestic roles in cooking from women to men against the social norm might result in men’s experience of psychosocial stress and adverse health outcomes [4, 21, 22]. However, socio-economic status might influence men’s or women’s attitudes towards cooking, thus generating different outcomes [19, 23]. College-educated men spent longer time cooking at home compared to men with less education [24], which could suggest their positive attitudes towards cooking. Men who had less pressure from work and life, i.e. at a high socio-economic status, may regard cooking as their leisure time [19]. However, intense workload might make women view cooking as stressful [23].

Evidence on cooking frequency and hypertension is limited, and gender difference is underexplored. Previous study suggested home prepared food could decrease risk of hypertension [9, 10], while some found longer time on cooking was associated with higher metabolic syndrome risks [25], and cooking oil fumes generated in the cooking process may increase hypertension risk [13]. In addition, switched gender roles in cooking might cause social stress, especially for men, which could lead to hypertension [4]. Therefore, the overall effect of cooking on hypertension is unclear and requires further exploration.

In this study, we aimed to examine whether cooking at home was associated with hypertension with consideration of gender differences from half a million Chinese population.

## Methods

### Study design and participants

We utilized the baseline data of the China Kadoorie Biobank (CKB) study conducted from 2004 to 2008 in China. Participants included adults aged 30 to 79 years from 10 geographically defined areas in China. These 10 areas were selected using criteria based on the following factors: local disease patterns, risk exposures, population stability, quality of death and disease registries, and economic development. The chosen areas covered an equal representation of rural (Gansu, Henan, Sichuan, Hunan, and Zhejiang) and urban (Harbin, Qingdao, Suzhou, Liuzhou, and Haikou) provinces, and approximately 44.6% all participants were from urban regions. In each region, permanent residents with no major disability were invited to participate. Potential participants were approached in person by community leaders or health workers. The estimated population response rate was about 30% (26–38% in the five rural areas and 16–50% in the five urban areas) [26]. A total of 512,891 individuals, representing approximately 30% of the total population of these 10 selected regions, completed interviewer-administered computerized questionnaires and clinical visits. During clinical visits, biophysical characteristics were measured, including height and weight, hip and waist circumference, bio-impedance, systolic and diastolic blood pressure (mmHg) (SBP/DBP) and lung function. Further details on how measurements were collected were described elsewhere [26].

### Exposure variable

The primary exposure of interest was cooking frequency. Participants were asked about their cooking frequency in present residences (daily, weekly, monthly, never/ rarely cook and no kitchen). To balance the sample sizes in each category and maintain comparability, we then defined cooking frequency as a 3-level categorical variable: never cooking (“never/rarely cook” and “no kitchen”), weekly or monthly cooking (“weekly cook” and “monthly cook”), and daily cooking (“daily cook”).

### Outcome variable

The primary outcome of interest was whether participants were hypertensive. Hypertension was a binary variable with 1 representing hypertensive and 0 representing non-hypertensive. Blood pressure was measured twice by trained staff using a digital sphygmomanometer (Omron UA-779), after participants had remained seated at rest for at least 5 minutes. If the difference between the two SBP measurements was greater than 10 mmHg, a third measurement was conducted; the mean value of the last two measurements was used for analysis. Regular calibration was made for all devices to ensure measurement consistency.

We considered participants to be hypertensive if they had a measured systolic/diastolic blood pressure (SBP/DBP) over the 140/90 mmHg threshold, referencing the 1999 World Health Organization/International Society of Hypertension (WHO/ISH) guidelines on the diagnosis of hypertension [27], or if they reported a diagnosis of hypertension by a physician.

### Other covariates

Other covariates in the analysis included demographic and socio-economic characteristics, self-rated health, lifestyle factors and household air pollution. Demographic and socio-economic characteristics included age, region (urban/rural), highest level of education (no education, primary school, middle/high school, college and above), household income (<5000yuan, 5000–19,999yuan, ≥20,000 yuan), marital status (married, other (widowed, separated/divorced, never married)) and occupation (agriculture and related, factory worker, clerk (i.e. administrator/manager, professional/technical, sales and service workers, self-employed and others), self-employed, unemployed (i.e. unemployed, retired and house wife/husband)). Self-rated health was also included to reflect general health status of participants (excellent, good, fair, poor). Lifestyle factors included metabolic equivalent task hours (MET-hours/day), BMI (kg/m2), smoking habits (never, occasional, regular), and alcohol use (never, occasional, regular). Household air pollution was assessed by using two variables as proxies: the presence of chimney (all stoves having chimneys, not all stoves having chimneys, no stoves having chimney), and stove kept slow-burning throughout the day (always, sometimes, never).

### Statistical analyses

We examined the distribution of baseline characteristics and hypertension status among groups of men and women with different cooking frequencies using descriptive statistics. One-way ANOVA (for continuous variables) and Chi square tests (for categorical variables) were used to compare the differences in baseline characteristics among men/women with different cooking frequencies. We estimated the associations between cooking frequency and hypertension in men and women respectively, using univariable and multivariable logistic regression models. The fully adjusted multivariable models were adjusted for all demographic and socio-economic characteristics, self-rated health, and lifestyle factors. In a sensitivity analysis, we adjusted for additional variables on household air pollution. Adjusted odds ratios (AORs) and 95% confidence intervals (95% CIs) were calculated.

To further explore the relationship between cooking frequency and hypertension among men and women, we conducted stratified analyses by all demographic and socio-economic characteristics using multivariable logistic regression models. Stratified analyses were adjusted for all covariates in fully adjusted models other than the stratification variable.

Two-sided *p* values < 0.05 were considered statistically significant. All statistical analyses were conducted using SAS software, version 9.4 (SAS Institute, Cary, North Carolina, USA).

## Results

Among the 512,891 Chinese participants included in this study, we found that while most women cooked daily (*N* = 254,198, 84.00%), only less than a quarter of men (*N* = 47,091, 22.40%) cooked daily, and the majority of men never cooked (*N* = 111,996, 53.27%) (see Table 1). Among women, compared with those who cooked monthly or weekly or those who never cooked, those who cooked daily tended to be older, have a lower education level, live in rural areas with a household income of less than 5000 Yuan, and work in agriculture and related fields. They generally had a lower MET, had chimney at home, and always kept their stoves slow-burning throughout the day. Whereas among men, a higher proportion of those who cooked daily lived in urban areas, were not married, had a household income of less than 5000 Yuan, were unemployed, had a lower MET, and never smoked. Higher prevalences of hypertension were observed in both women and men who cooked daily, compared with those who cooked monthly or weekly or those who never cooked. All baseline characteristics differed significantly among men/women with different cooking frequencies (*p* values < 0.001).

**Table 1:** Socio-demographic and lifestyle characteristics by gender

	Women (*N* = 302,632)*			Men (*N* = 210,259)*		
	Daily Cooking *n* = 254,198	Weekly or Monthly Cooking *n* = 29,263	Never Cooking *n* = 19,171	Daily Cooking *n* = 47,091	Weekly or Monthly Cooking *n* = 51,172	Never Cooking *n* = 111,996
Age, years (SD)	51.27 (10.33)	49.71 (10.79)	48.81 (11.50)	52.18 (10.96)	51.53 (10.68)	53.64 (10.90)
Urban, %	41.36	66.32	53.65	58.70	56.31	31.16
Highest level of education, %						
Primary school and below	58.61	47.10	46.28	38.91	38.48	45.33
Middle/high school	37.77	44.69	43.95	51.42	51.92	48.33
College and above	3.61	8.20	9.76	9.66	9.60	6.34
Household income, %						
< 5000 yuan	11.24	4.25	4.87	12.05	6.08	9.53
5000–19,999 yuan	51.52	37.45	35.81	42.25	38.74	49.23
≥ 20,000 yuan	37.24	58.30	59.32	45.70	55.18	41.24
Marital status, %						
Married	88.13	90.47	88.87	83.85	95.32	95.60
Other	11.87	9.53	11.13	16.15	4.68	4.40
Occupation, %						
Agriculture and related	44.61	20.04	18.10	30.45	33.60	53.35
Factory workers	8.03	23.21	25.88	23.90	25.42	14.26
Clerk	7.30	17.15	18.30	12.36	14.59	10.60
Self employed	1.70	3.80	5.86	2.39	3.79	3.85
Unemployed	38.36	35.80	31.86	30.90	22.60	17.94
Self-rated health*, %*						
Excellent	15.66	15.79	20.77	21.61	19.75	19.42
Good	27.09	29.01	28.33	27.22	28.80	30.30
Fair	45.85	45.07	38.36	41.36	43.67	41.22
Poor	11.40	10.13	12.54	9.80	7.78	9.06
Hypertension, %	33.04	28.60	29.79	39.88	35.99	35.03
MET, hours/day (SD)	20.09 (12.28)	22.26 (14.35)	21.99 (15.67)	21.02 (13.97)	23.00 (15.06)	22.00 (15.88)
BMI, kg/m2 (SD)	23.86 (3.47)	23.54 (3.36)	23.60 (3.47)	23.69 (3.34)	23.63 (3.24)	23.23 (3.20)
Smoking, %						
Never	94.85	95.92	94.53	16.58	13.60	13.89
Occasional	2.71	2.35	3.09	26.50	26.00	23.00
Regular	2.44	1.73	2.38	56.92	60.40	63.11
Alcohol, %						
Never	63.56	66.47	66.38	22.70	21.60	25.87
Occasional	34.01	30.92	30.27	34.97	36.91	39.21
Regular	2.43	2.61	3.35	42.33	41.49	34.92
Household have chimney, %	83.97	82.90	75.33	77.81	79.35	73.11
Stove kept slow-burning throughout the day, %						
Always	31.29	11.35	14.77	21.98	21.43	36.00
Sometimes	14.92	7.18	6.05	8.42	10.50	16.75
No	53.79	81.47	79.18	69.60	68.07	47.25

*All baseline characteristics differed significantly among men/women with different cooking frequencies (*p* values < 0.001)

We found significant yet different associations between cooking frequency and hypertension in men and women, respectively (see Table 2). Among men, compared to those who never cooked, those who cooked weekly or monthly had a higher odds of hypertension (AOR: 1.05, 95% CI: 1.02–1.07), and those who cooked daily had an even higher odds (AOR: 1.09, 95% CI: 1.06–1.11), suggesting a dose-effect relationship between cooking frequency and hypertension. In contrast, increased cooking frequency has a protective effect against hypertension among women. Those who cooked weekly or monthly and those who cooked daily had lower odds of hypertension than those who never cooked (AOR: 0.94, 95% CI: 0.89–0.99; AOR: 0.96, 95% CI: 0.92–0.99 respectively). We adjusted for household air pollution in an additional sensitivity analysis and obtained similar results (results not shown).

**Table 2:** The associations between cooking frequency and hypertension in men and women

	Women	Men
	Unadjusted OR (95%CI)	
Never Cooking	1	1
Weekly or Monthly Cooking	0.94 (0.91,0.98)	1.04 (1.02,1.07)
Daily Cooking	1.16 (1.13,1.20)	1.23 (1.20,1.26)
	Adjusted OR (95%CI)^a^	
Never Cooking	1	1
Weekly or Monthly Cooking	0.94 (0.89,0.99)	1.05 (1.02,1.07)
Daily Cooking	0.96 (0.92,0.99)	1.09 (1.06,1.11)

^a^Adjusted for age, region (urban/rural), highest level of education, self-rated health, occupation, marital status, household income, BMI, MET, alcohol, smoking

**Fig. 1:**
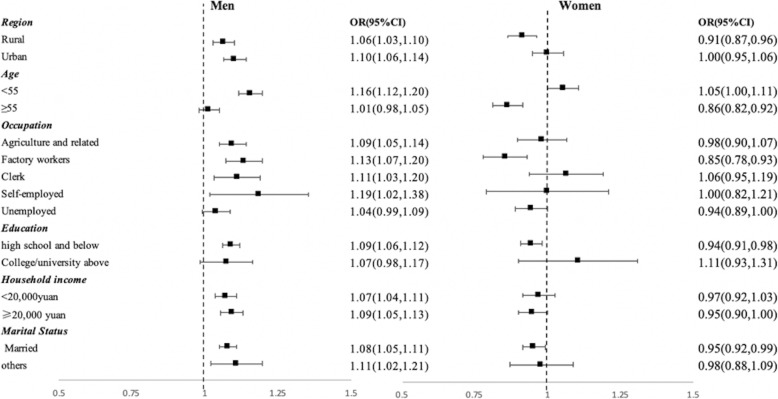
The association between daily cooking and hypertension stratified by socio-economic status in men and women. Note: Reference group: never cooking; Adjusted for all covariates in the fully adjusted model other than the stratification variable

**Fig. 2:**
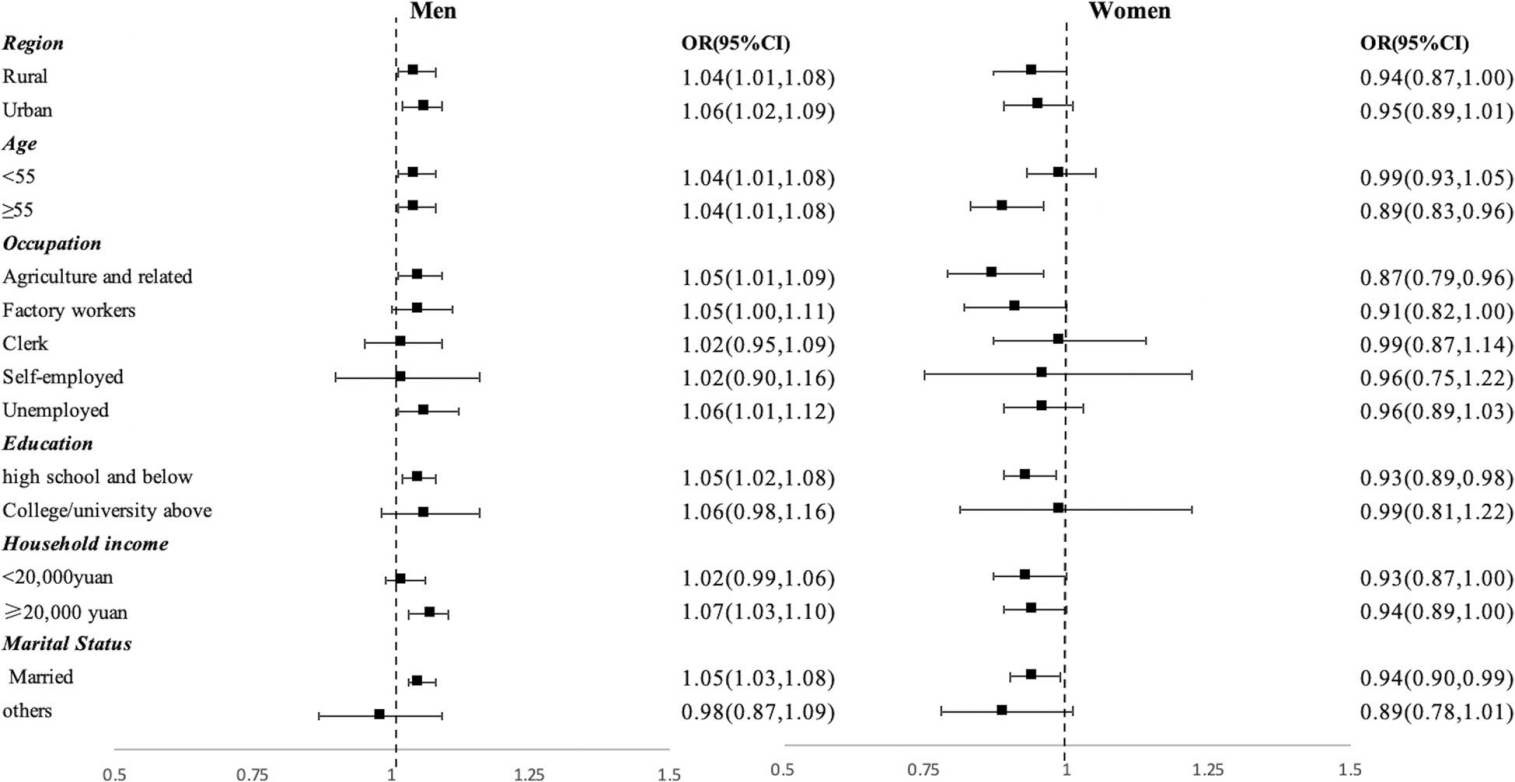
The association between weekly or monthly cooking and hypertension stratified by socio-economic status in men and women. Note: Reference group: never cooking; Adjusted for all covariates in the fully adjusted model other than the stratification variable

Further, we observed similar results in stratified analyses by demographic and socio-economic status in men and women, respectively: daily, and weekly/monthly cooking was associated with a higher odds of hypertension in men and a lower odds of hypertension in women (see Fig. 1). Among men, significant associations between daily cooking and hypertension were observed in most socio-demographic categories except for those over 55 years of age (AOR: 1.01, 95%CI: 0.98–1.05), those who were unemployed (AOR: 1.04, 95%CI: 0.99–1.09), and those who had a college/university education or above (AOR: 1.07, 95%CI: 0.98–1.17). In women, daily cooking was generally protective against hypertension, especially for those who lived in rural areas (AOR: 0.91, 95%CI: 0.87–0.96), those over 55 years of age (AOR: 0.86, 95%CI: 0.82–0.92), those who were factory workers (AOR: 0.85, 95%CI: 0.78–0.93), unemployed (AOR: 0.94, 95%CI: 0.89–1.00), married (AOR: 0.95, 95%CI: 0.92–0.99), had a high school education or below (AOR: 0.94, 95%CI: 0.91–0.98), and had a household income higher than 20,000 yuan (AOR: 0.95, 95%CI: 0.90–1.00). However, we did not observe protective effects in other groups of women, such as those living in urban areas, those who had high education levels, those with low household income and those who were not married. Besides, Younger women in fact had a higher odds of hypertension from cooking, but the magnitude is smaller than their male counterparts (AOR: 1.05, 95%CI: 1.05–1.11 vs. AOR:1.16, 95%CI: 1.12–1.20). Stratification results for weekly and monthly cooking were slightly different from those of daily cooking (see Fig. 2). The positive association of weekly or monthly cooking with hypertension was not observed among men who were clerks (AOR: 1.02, 95%CI: 0.95–1.09), who were self-employed (AOR: 1.02, 95%CI: 0.90–1.16), who had college and above education (AOR: 1.06, 95%CI: 0.98–1.16), who had a household income less than 20,000 yuan (AOR: 1.02, 95%CI: 0.99–1.06) and who were not married (AOR: 0.98, 95%CI: 0.87–1.09). The negative association of weekly or monthly cooking with hypertension was not observed among women who were younger than 55 years (AOR: 0.99, 95%CI: 0.93–1.05), who were clerks (AOR: 0.99, 95%CI: 0.87–1.14), who were self-employed (AOR: 0.96, 95%CI: 0.75–1.22), who were unemployed (AOR: 0.96, 95%CI: 0.89–1.03), who had college and above education (AOR: 0.99, 95%CI: 0.81–1.22), and who were not married (AOR: 0.89, 95%CI: 0.78–1.01).

## Discussion

In our analysis of 512,891 participants in a population-based cross-sectional study in China, we found that men who ever cooked (including daily cooking and monthly or weekly cooking) had higher odds of hypertension compared with those who never cooked, while a negative association was observed in women. Socio-economic status including occupation, household income, education and region further modified the effect of daily cooking on hypertension among men and women, respectively.

Few previous studies have examined the relationship between cooking frequency and hypertension, and gender differences in the association is under-explored. Home meal preparation was believed to generate better health outcomes [5, 28]. A study in Taiwan found that higher cooking frequency was favorable to survivorship among Taiwanese especially for women [29]. Home cooking involvement is associated with less healthy eating pattern for boys not girls [30], which is comparable to our results. However, SWAN study found that women in US who spent more time preparing and cleaning up meals had higher metabolic syndrome risks, and postulated that longer food preparation time at home did not guarantee healthier food [25]. Cooking at home could also be detrimental to health due to harmful chemicals emitted in the process [15, 31, 32]. Previous studies have shown that household fuel fumes, including cooking oil fumes, could elevate blood pressure [13], which was not found in our study. After adjusting for household air pollution, cooking was still harmful to men, and protective towards women in the present study.

The observed different associations between cooking frequency and hypertension in men and women might result from different food preferences among men and women, as people generally cook meals according to their preferred flavor. Studies have shown that men and women prefer different types of diet, with women being more likely than men to avoid high-fat food, have higher intake of fruits and fiber, and limit salt intake [33]. Men were also more positive about the nutrition value of convenience food [34] which was actually less healthful. Women assigned more importance to healthy food attributes (i.e. organic, local, GM-free) when choosing food sources [35]. High-fat and high-salt diet is known to be an important risk factor for hypertension [36, 37]. Consequently, the observed difference in hypertension could result from different diet preferences by women and men. Furthermore, because of the wide definition of cooking, the observed gender differences might also rise from different perceptions of cooking between men and women [34, 38, 39]. Men might view preparing ready-to-eat-meals as a way of “cooking”, while women might consider preparing a full meal as “cooking”. The different perceptions might also explain the stronger association of cooking with hypertension prevalence in men [38].

Our finding of the gender difference might also be explained through social pathways. As was suggested by the Taiwanese study, the observed gender difference in the association between cooking frequency and survivorship might result from the stereotype of female being the domestic role of cooking [29]. Housework is typically viewed as women’s work [19]. Some women took cooking as their responsibilities, and might enjoy cooking and consider it as a way to spend their leisure time and reduce stress, resulting in a reduced hypertension odds [4, 40, 41]. Men, on the other hand, tend to view housework as “women’s work” [19, 42], and undertaking such tasks may be internalized or seen by their peers as a threat to their masculine identity [43]. If men were pressured to cook, they would be more likely to experience stress, elevating hypertension risks over time [4].

Furthermore, socio-economic factors may modify the effect of cooking frequency on hypertension as was shown in the present study. We found that daily cooking did not have a protective effect against hypertension among women who lived in urban areas, had high education levels, were not married and were relatively young (< 55 years old). Social stress from work and life faced by these women might make the cooking process stressful, thus counteracting the protective effect of cooking against hypertension in women [4, 23, 40]. We did not observe a positive association between daily cooking and hypertension among men who were older (≥55) and unemployed. Men who have less pressure from work and life may also regard cooking as their leisure time and enjoy cooking, and thus also have a lower odds of hypertension [19].

### Strengths and limitations

To our knowledge, this is the first study that examines the gender difference in the association between cooking frequency and hypertension using a large sample size of the general Chinese population. By conducting stratified analyses by socio-economic status, we were able to explore potential modifiers of the main association in men and women, respectively. There are a few limitations to our study. First, there may be other gender-related confounders that were unmeasured or not adjusted for in our analyses. Future studies should aim to further explore factors that may contribute to the observed gender differences, for example, diet preferences or social stress. Second, our capacity to establish a causal relationship is limited due to the cross-sectional study design. We could not establish time sequence of cooking behaviors and hypertension diagnosis. Reverse causality may exist as people with hypertension might alter their cooking behaviors. Future longitudinal studies are needed to further explore the observed relationships.

## Conclusion

In a population-based Chinese study of 512,891 participants, we found that compared to those who never cooked, men who had ever cooked had an elevated odds of hypertension, while women who had ever cooked had a reduced odds. The mechanisms that underlie such associations might be gender differences in food preferences and attitudes towards cooking. Interventions should promote healthy food preparation in home cooking and also address psycho-social stress related to cooking in men and women.

## Data Availability

The datasets used and/or analyzed during the current study are available from the corresponding author on reasonable request.

## Abbreviations

95% CIs: 95% confidence intervals
AOR: Adjusted odds ratios
CKB: China Kadoorie Biobank
DBP: Diastolic blood pressure
MET: Metabolic equivalent task hours
SBP: Systolic blood pressure
WHO/ISH: World Health Organization/International Society of Hypertension
